# Electronic cigarettes in mental health settings - solving a conundrum?†

**DOI:** 10.1192/pb.bp.114.047431

**Published:** 2014-10

**Authors:** Elena Ratschen

**Affiliations:** 1 University of Nottingham, Nottingham

## Abstract

Electronic cigarettes (e-cigarettes), have recently been the focus of much attention and debate. This article attempts to highlight their relevance and potential importance for mental health settings, with a focus on in-patient units. To do so, the complexities involved in smoking among people with mental disorder, the debate surrounding e-cigarettes, and their potential to be utilised as a smoking cessation or temporary abstinence aid in the context of smoke-free policies and new National Institute for Health and Care Excellence guidance for smoking cessation in mental health settings, will be discussed and synthesised below.

## Smoking, smoke-free policies and mental disorder

The importance of addressing tobacco smoking among people with mental disorder is now well recognised, and increased research activity, policy initiatives and widespread endorsement^1-3^ raise hopes that, in the UK, progress and change are underway. As presented in more detail than this article allows, elsewhere the evidence shows that people with mental disorder are not only substantially more likely to smoke, but also smoke more heavily than people without a mental disorder,^4,5^ thus consuming over 40% of all cigarettes smoked in the UK^6^ - a figure as staggering as it is worrying. As one would expect, morbidity and mortality because of smoking-related conditions, such as cardiovascular and respiratory disease, are elevated accordingly, adding to the burden of excess premature deaths and life years lost as a result of other causes in this disadvantaged population.^1^ Most worrying, however, is the circumstance that despite this and the fact that people with mental disorder can be as motivated to address their smoking as people without,^7^ smoking remains deeply entrenched in the culture of mental health settings, and is rarely addressed in clinical practice - often because of the belief that it fulfils therapeutic functions with regard to certain symptoms of some mental disorders, and out of reluctance to interfere with patients’ ‘choice to smoke’ (although the term ‘choice’ in the context of using a substance as addictive as heroin may arguably be ill chosen). There are other reasons for the persistent failure to support smokers with mental disorders to equally good standards as smokers without that are too many and too complex to discuss here in detail and that are documented elsewhere.^8-11^

Be that as it may. The recently published Royal College of Physicians and Royal College of Psychiatrists’ report on smoking and mental disorder,^1^ as well as the National Institute for Health and Care Excellence (NICE) guidance on smoking cessation in secondary care including mental health settings^2^ mark, one would hope and trust, the dawn of a new area. Mental health trusts are now expected to provide prompt, comprehensive and evidence-based on-site support to help smokers quit or maintain temporary abstinence in completely and strictly smoke-free mental health settings. It is important to note that this expectation goes decidedly beyond the standard most mental health trusts fulfil in the context of the smoke-free legislation that came into force under the Health Act 2006. Although many mental health trust policies drafted to comply with the Act stipulate smoke-free trust premises, the reality tends to look different: although indoor spaces are now, with greater or lesser success, kept smoke free, it is the norm to see patients smoke on trust grounds, near entrances, in courtyards or on balconies, and to observe staff spending time (that could and should arguably be used for more constructive purposes) facilitating smoking breaks (or ‘fresh air breaks’, as they are sometimes called) for in-patients who are smokers.^9,12^

Anecdotally, the discontent of staff and patients with how smoking and not smoking are currently being handled in many mental health settings appears ubiquitous. Less clear is what to do about it - and how to fulfil the expectations of the new NICE guidance, which are, compared with the current status quo, high and, some would argue, in places short of being accompanied by clear practice recommendations that would enable trusts to fulfil them in a straightforward manner. Although there is probably good reason to argue that policies and procedures to deal with alcohol on in-patient units could be usefully extrapolated to cover smoking, seeing as both are legal substances that are not to be consumed on trust premises, it seems that the comparison or even likening of the two is not always perceived as wholly intuitive, and so the call for clear guidance on how to deal with smoking in particular remains. The problem is that a strong evidence base that would confidently suggest how best to eliminate smoking and treat tobacco dependence in mental health in-patient settings (where no exemptions from the policy are allowed) is missing. There is some evidence, but it is limited,^13-15^ sometimes referring to small studies with particular parameters (such as high secure forensics) and not always taking into account the complexities some mental health settings are facing - one size does, as so often, not necessarily fit all. It also indicates that more research into effective ways of supporting acutely ill in-patients who smoke and have mental disorder to quit or temporarily abstain from smoking is needed, and that existing approaches need to be tailored and adapted to the setting.^2^ In short, creative ways of thinking are required, and the author of this article believes that e-cigarettes could play a part in it.

### E-cigarettes in summary

Electronic cigarettes are devices that deliver nicotine through a vapour (not smoke) that, apart from the nicotine itself, contains propylene glycol, glycerine, sometimes flavours and a range of other by-products of the vaporising process, but none of the highly toxic components found in tobacco smoke.^16,17^ Electronic cigarettes typically resemble a normal cigarette in appearance (although new types of e-cigarettes can use different designs), but contain a battery, a cartridge with a nicotine solution and a heating device that is triggered by drawing air through the device to produce vapour. Although the vapour is not as pure as the vapour created by licensed medical devices (such as the nicotine replacement therapy (NRT) inhaler), it is evidently much less hazardous than tobacco smoke, marking e-cigarettes as devices able to provide nicotine in a way that mimics many of the behavioural components of smoking, with relatively minimal risks.^16^ As nicotine is the addictive substance in tobacco smoke, but smokers are killed by other toxic components, the potential benefit of e-cigarettes for cigarette smokers is obvious and has been described as vast in terms of improving public health.^16^ Risks of exposure to second-hand e-cigarette vapour are yet to be assessed.

However, concerns have been raised with regard to potential hazards of e-cigarette use, summarised and discussed comprehensively in a recent publication by members of the UK Centre for Tobacco and Alcohol Studies.^16^ One area of scepticism refers to the lack of data on the safety of e-cigarettes and to how these devices should be regulated to ensure that their public health potential is maximised, while risks are minimised. Reflecting these concerns, e-cigarettes, which are currently licensed under general product safety regulations, are to be regulated either by the UK Medicines and Healthcare Product Regulatory Agency (MHRA) or by the European Union tobacco products directive in the near future. In the interim, e-cigarettes remain available to users, while manufacturers work towards meeting the higher purity and delivery standards of medicinal product licensing, with the first of these products expected to be available from the end of 2014. Other areas of concern are summarised and discussed in detail elsewhere and include arguments referring to e-cigarettes being a potential gateway into tobacco smoking, undermining the progressive denormalisation of smoking in society, and being used by the tobacco industry to promote cigarettes. At the end of the day and of a debate that seems to be fuelled with the emotion of strongly held convictions, however, three facts remain: particularly if regulated to high product and delivery standards, e-cigarettes are definitely far less hazardous than normal cigarettes; they have the potential to help smokers quit or abstain from tobacco use; and - in contrast to NRT and other pharmacotherapy for smoking cessation - they are proving very popular and acceptable, with their use increasing dramatically over time and more than 15% of smokers reporting having tried them.^16^

## E-cigarettes, smoke-free policy and mental disorder

How could e-cigarettes be of particular value in mental health settings? Many trusts are currently confronted with what must feel like a conundrum: there is all the good will to make mental health settings completely smoke free, to offer smokers gold standard support, to work towards closing the gap between smokers with mental disorder and smokers without by changing the persistent smoking culture in these settings - and yet, there is not much wisdom as to how to achieve this. It is true that, generally speaking, treatments for tobacco dependence that work for the general population (i.e. a combination of pharmacotherapy and behavioural support) also work for people with mental illness,^18^ although certain caveats with regard to the use of bupropion and varenicline apply.^1,18^ However, the evidence supporting this stems from clinical trials that recruited particular subgroups of smokers with mental disorder: those who were stable, usually living in the community (a standard inclusion criterion for research in this area) and who were, above all, willing to address their smoking and consent to being part of smoking research in the first place. Every clinician working with people with mental disorder on acute adult mental health in-patient units on a daily basis knows that there is a huge difference between their patients and the recruits in these studies.

Pilot research on in-patient units has demonstrated that the mantra of offering NRT and specialist smoking cessation advice in everyday practice results in modest success rates in terms of service uptake and smoking cessation or abstinence.^14^ Nicotine replacement therapy products, although generally effective and doubling success rates for smoking cessation compared with no use of pharmacotherapy,^19^ are undoubtedly products of limited popularity - whereas it should be noted that mental health professionals’ often sceptical stance, probably based on misconceptions with regard to their use in the context of mental disorder,^20^ do a disservice to the products and needs to be addressed. It is, however, conceivable that to smokers admitted to a mental health in-patient unit in an acute state of crisis, NRT may seem like yet another medication, and one whose administration may not be entirely intuitive or without pitfalls. The need to administer frequently and regularly if a heavy smoker (with likely underdosing resulting in limited effectiveness and quick dissatisfaction with the product) and potential difficulties associated with use, such as chewing techniques for the gum, side-effects of the nasal spray and unpleasant taste of the products, may just be enough to prevent success in acute mental health settings - where patients’ motivation and resources to address smoking are likely to be fluctuating. It is probably fair to say that what mental health trusts are currently facing - albeit undoubtedly and rightly in the name of health promotion and equality - is the somewhat daunting prospect of enforcing entirely smoke-free trust premises, while trying to offer the majority of their patients treatment for cessation or temporary abstinence that they might not want to use. To complicate matters and intensify apprehensions that may or may not prove justified, there are added concerns over introducing inequalities between formal and voluntary patients in terms of the ability to access tobacco (off trust premises), over the loss of voluntary patients (feared and anecdotally reported to become reluctant or unwilling to admit themselves to in-patient units when not able to smoke), and over escalations relating to strict policy enforcement (for example through body searching for smoking paraphernalia on (re-)entering the ward).

Would it not be wonderful if there was something on offer that people really liked? Something they could use in the same way they use cigarettes, that was, however, relatively safe (and appropriately licensed), something they might find at least quite attractive and had perhaps used in the past, that substituted for their normal cigarettes for their time on the ward, and that may, as a by-product, result in substantial reduction or cessation of tobacco use in the long term? Perhaps e-cigarettes could be just that. Although still limited, the evidence for the general population suggests that e-cigarettes could serve as an effective cessation aid,^21^ and a pilot study of smokers with schizophrenia who were not willing to quit showed that e-cigarette use substantially decreased cigarette consumption over 12 months, without significant side-effects or a negative impact on the symptoms of schizophrenia.^22^ And would it not seem promising to inform a patient on admission (and before, where possible) that tobacco use is not allowed on trust premises, but that they could use e-cigarettes instead, should they prefer or would like to add it to the offer of standard smoking cessation treatment with NRT and behavioural support - more promising than seizing cigarettes and offering standard treatment only? Bearing in mind the circumstance that the risks of exposure to second-hand e-cigarette vapour have not been thoroughly assessed, patients could be asked to use e-cigarettes in their private rooms or outdoors only. Both formal and voluntary patients could use the devices, through greater acceptability and popularity perhaps at least partly removing the issue of voluntary patients continuing tobacco smoking off trust premises and re-entering the ward smelling of cigarettes, and perhaps carrying paraphernalia with them. Granted, not all challenges or potential problems would suddenly be solved. For example, risk assessments would need to consider how to handle issues relating to devices that require regular charging would need to be addressed (perhaps by prescribing the use of e-cigarettes with non-rechargeable batteries only?), or how to address any potential fire risks.

Commissioning of (licensed) products along the lines of NRT is another example of discussions likely to ensue. Still, on reflection, short of hailing e-cigarettes as revolutionary devices for mental health in-patient settings, it is probably reasonable to state that they could change the way smoking and smoke-free policies are handled, and help trusts and clinicians with solving the conundrum they find themselves confronted with. It certainly seems worth a try, and worth some research. Indeed, some trusts in the country are already incorporating e-cigarettes in their smoke-free policies, and it will be important to share and learn from each others’ experience along the way of what will hopefully become a major avenue towards health promotion in mental health settings.
